# Pakistani women's use of mental health services and the role of social networks: a systematic review of quantitative and qualitative research

**DOI:** 10.1111/hsc.12305

**Published:** 2015-11-22

**Authors:** Dharmi Kapadia, Helen Louise Brooks, James Nazroo, Mark Tranmer

**Affiliations:** ^1^ The Cathie Marsh Institute for Social Research (CMIST) The University of Manchester Manchester UK; ^2^ School of Nursing, Midwifery and Social Work The University of Manchester Manchester UK

**Keywords:** ethnic minorities, mental health services, Pakistani, social support, stigma

## Abstract

Pakistani women in the UK are an at‐risk group with high levels of mental health problems, but low levels of mental health service use. However, the rates of service use for Pakistani women are unclear, partly because research with South Asian women has been incorrectly generalised to Pakistani women. Further, this research has been largely undertaken within an individualistic paradigm, with little consideration of patients’ social networks, and how these may drive decisions to seek help. This systematic review aimed to clarify usage rates, and describe the nature of Pakistani women's social networks and how they may influence mental health service use. Ten journal databases (ASSIA, CINAHL Plus, EMBASE, HMIC, IBSS, MEDLINE, PsycINFO, Social Sciences Abstracts, Social Science Citation Index and Sociological Abstracts) and six sources of grey literature were searched for studies published between 1960 and the end of March 2014. Twenty‐one studies met inclusion criteria. Ten studies (quantitative) reported on inpatient or outpatient service use between ethnic groups. Seven studies (four quantitative, three qualitative) investigated the nature of social networks, and four studies (qualitative) commented on how social networks were involved in accessing mental health services. Pakistani women were less likely than white (British) women to use most specialist mental health services. No difference was found between Pakistani and white women for the consultation of general practitioners for mental health problems. Pakistani women's networks displayed high levels of stigmatising attitudes towards mental health problems and mental health services, which acted as a deterrent to seeking help. No studies were found which compared stigma in networks between Pakistani women and women of other ethnic groups. Pakistani women are at a considerable disadvantage in gaining access to and using statutory mental health services, compared with white women; this, in part, is due to negative attitudes to mental health problems evident in social support networks.

**What is known about this topic**

- Pakistani women in the UK have high levels of mental health problems, but their rate of mental health service use has not been clearly established.
- Social networks can increase or decrease service use, but this association has not been investigated in the UK.

**What this paper adds**

- Pakistani women were less likely than white women to use specialist mental health services, but were no less likely to consult general practitioners for mental health problems.
- High levels of mental health stigma were evident in Pakistani women's social networks and acted as a deterrent to using services, but no studies were identified that compared stigma across ethnic groups.

## Introduction

The Delivering Race Equality programme (Department of Health, 2005) aimed to provide equitable, non‐racist mental health services to people in England and Wales. Its success was limited and after its end in 2010, ethnic inequalities in mental health service provision remained (Community and Mental Health Team: Health and Social Care Information Centre, 2014a). South Asian (Pakistani, Indian and Bangladeshi) women are one group for whom inequalities (low rates of usage of mental health services) are evident (C. Cooper *et al*. 2013, J. Cooper *et al*. 2010). Within this group, Pakistani women may be particularly disadvantaged, as they have high levels of mental illness (Gater *et al*. 2009, Chaudhry *et al*. 2012), but low levels of service use. However, there is little robust evidence, as typically the rates of usage for Pakistani women have been inferred from South Asian women. It is not appropriate to do this, as there are indications that Pakistani women have higher mental illness rates than Indian and Bangladeshi women (Nazroo 2001, Weich *et al*. 2004), but lower usage of mental health services than Indian women (Care Quality Commission & National Mental Health Development Unit, 2010, 2011).

Pakistani women may have low mental health service use because they are less likely to be referred to specialist mental health services (Burman *et al*. 2002) and the National Health Service (NHS) may be inadequate in addressing religious, cultural and language needs (Chew‐Graham *et al*. 2002, Bowl 2007). Further, Pakistani women may be fearful that confidentiality may not be maintained (Gilbert *et al*. 2004). These reasons reflect the tendency of research on mental health service use to focus on how individuals (patients) in conjunction with systems (NHS) drive the outcomes of mental healthcare pathways. Therefore, unsurprisingly, the candidacy model (Dixon‐Woods *et al*. 2005), has been the theoretical model of choice for many recent reviews of health service usage (Garrett *et al*. 2012, Lamb *et al*. 2012). This model largely ignores the social aspect of help‐seeking; the way in which decisions and actions are influenced by the people closest to us (Pescosolido 1992, 2011, Thornicroft 2006). Social networks may be particularly important for those groups who are alienated from mental health service systems, both in terms of their content (the people in them – friends, family), and their function (provision of support, exchange of information about illness and services). Social support within social networks may be protective, by reducing the propensity to develop mental illness, and for people who have developed mental illness, social networks may act as a substitute for services (Gourash 1978). Certainly, research in the US, Netherlands and Puerto Rico has shown that people were less likely to use mental health services if high levels of support were being provided within networks (Pescosolido *et al*. 1998, Ten Have *et al*. 2002, Maulik *et al*. 2009). However, in the UK, very little attention has been paid to the influence (either positive or negative) of the content and function of social networks on the usage of mental health services. This is an important omission, as research from other countries suggests that the explanations for low rates of mental health service use could be expanded upon and improved with reference to the content and function of social networks. In order to clarify the rates of usage of mental health services of Pakistani women, the nature of their social networks and the possible influence of social networks on mental health service use, the review answered these questions:

1. How does the usage of mental health services for Pakistani women in the UK compare with women from other ethnic groups?
2. What is the nature of Pakistani women's social networks and how does this compare with women from other ethnic groups?
3. What are the reasons for the mental health services usage patterns of Pakistani women and are social networks involved in the help‐seeking and access process?

## Methods

The review included quantitative and qualitative research studies. Mixed‐methods studies were not excluded, but there were not any mixed‐method studies that were deemed to be of sufficient quality to be included. Reviews incorporating quantitative and qualitative research have become more widely used in the social sciences (Evidence for Policy and Practice Information and Co‐ordinating Centre (EPPI‐Centre), 2010). For this review the data from studies were analysed thematically within each research question. It was not the intention of the review to generate a new theory of access to mental healthcare services, as there have been many studies that have already looked at this in‐depth, conceptually (Pescosolido 1992, Dixon‐Woods *et al*. 2005). Instead the review aimed to clarify the mental health usage rates for Pakistani women, and establish whether social networks are involved in the help‐seeking process.

### Inclusion and exclusion criteria

The inclusion criteria were: studies published from 1960 up to the end of March 2014, pertaining to Pakistani or South Asian women, on the subject of either access to, or usage of, mental health services or the nature of social networks, conducted in the UK and written in English. Studies from other countries were excluded due to the differing migration histories, socioeconomic positions and mental illness rates of Pakistani women in other countries. Papers that were theoretical in nature were excluded. Studies were excluded if they investigated access to child and adolescent mental health services, as the help‐seeking process that parents undertake on behalf of children is not directly comparable to the process in adult women. Papers related to dementia or learning disability services were excluded for similar reasons. Finally, studies investigating antidepressant or psychotropic medication use in Pakistani women that did not contain an element on access to services were also excluded.

### Study selection

A list of search terms was compiled by DK and JN, by drawing upon other systematic reviews in this area and the authors’ knowledge of previous research. Databases were searched for published journal articles, and a range of websites that indexed health and social care studies were searched to identify grey literature (see Table 1).

**Table 1:** Sources used in the review

Type of source	Databases
Electronic Databases (peer reviewed articles)	Applied Social Sciences Index and Abstracts (ASSIA) Cumulative Index to Nursing and Allied Health Literature (CINAHL Plus) EMBASE Health Management Information Consortium (HMIC) International Bibliography of the Social Sciences (IBSS) MEDLINE PsycINFO Social Sciences Abstracts Social Sciences Citation Index Sociological Abstracts
Grey Literature	OpenGrey Social Care Online Index to Theses Electronic Theses Online Services (ETHOS) The Health and Social Care Information Centre Website (HSCIC) Association of Health Observatories Website

**Figure 1:**
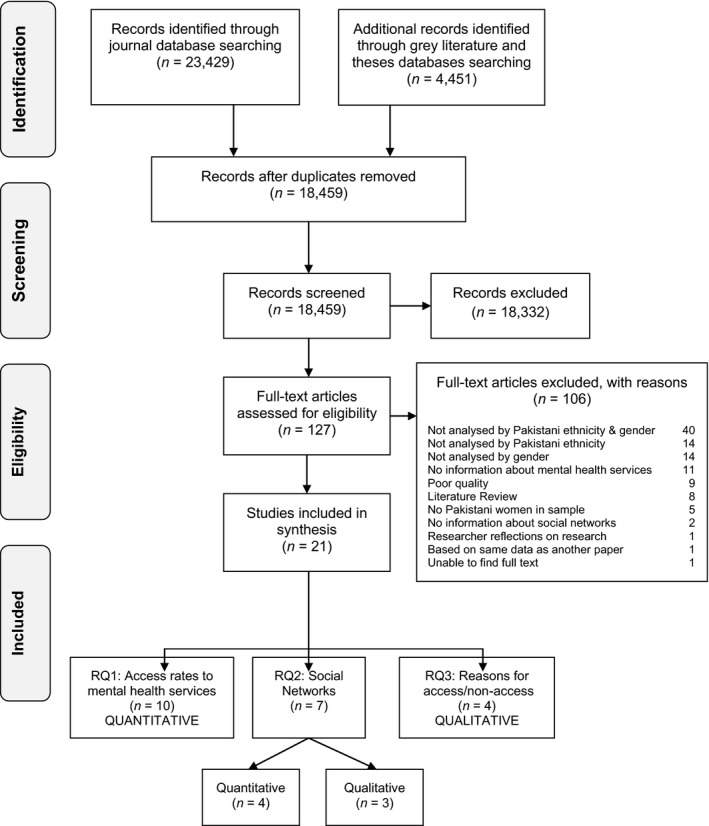
The Review Process of inclusion and exclusion of studies using PRISMA reporting (Moher *et al*. 2009).

Initial searches were tested in Medline and revised (see Box 1). Searches were adapted for each database. The Health and Social Care Information Centre (HSCIC) and Association of Health Observatories websites were searched manually. Searches were undertaken in April 2014. The searches yielded 27,880 papers. Results were imported into EPPI‐Reviewer 4 (Thomas *et al*. 2010) and the duplicate process was carried out, leaving 18,459 documents. Screening was undertaken by DK, after which, the full texts of 127 papers were reviewed. A PRISMA (Moher *et al*. 2009) diagram of the screening and eligibility process is shown in Figure 1.

Box 1 **Search terms for the review**

**Table:** 

Mental Health OR mental illness OR health service* OR healthcare disparit* OR health disparit* OR health equit* OR health inequit* OR health equal* OR health inequal* OR Health Care Services* OR Health Care Utilization* OR psychiatr* OR Health Care Psychology* OR access* OR health access* OR healthcare access* OR care path* OR help seek* OR service barrier* OR barrier to service* OR social network OR family network OR Social Support OR family support OR network analysis OR support network OR social capital AND ethnic* OR south asia* OR asian* OR pakistan* OR rac* OR Muslim* OR bme* OR minorit* AND uk* OR united kingdom* OR britain* OR Great Britain OR England

### Critical appraisal

Each of the 127 papers was critically appraised by DK and HB. Disagreements on inclusion (*n* = 5/127), were resolved by JN. Different quality assessment tools were used for each methodologically distinct study: for quantitative papers, the Study Quality Tool (Zaza *et al*. 2000) was used; for qualitative papers, the Critical Appraisal Skills Programme (CASP) Qualitative Checklist (CASP, 2014a); for mixed‐methods papers, the Mixed Methods Appraisal Tool (MMAT) (Pluye *et al*. 2011) and for systematic reviews, the CASP Systematic Review Checklist (CASP, 2014b). These tools were used as guides to assess the quality of the studies on which judgements were made about their inclusion. Papers that were appraised as poor on research design, inappropriate in the choice of methods or lacking robust analysis were excluded. During critical appraisal, posters and conference paper abstracts were excluded, but where possible, published papers referring to presentations were sought out. It was not possible to find one thesis that explored the health needs of Asian women in Manchester, despite making enquiries with the awarding university and the author.

At this stage, 106 papers were excluded (see Figure 1) as they were irrelevant to the research questions. Most papers (64%) were excluded because they did not analyse data by Pakistani ethnicity, gender or both. Nine papers (8%) were excluded due to poor quality. Six papers that documented the results of the Count me in Censuses 2005 to 2010 were included, despite providing estimates of mental health inpatient use that included people who were under the age of 18. This was because those aged under 18 years only constituted between 1% and 2.9% of inpatients in the years 2005–2010. The remaining 21 papers were categorised according to which research questions they addressed. Papers were published between 1999 and 2013, except one (published in 1977). Ten of these related to research question 1 (comparison of rates of use of mental health service between Pakistani women and women from other ethnic groups) and were quantitative in nature. Seven papers were relevant to research question 2 (the nature of Pakistani women's social networks); three were qualitative and four quantitative. Data were synthesised separately for quantitative and qualitative studies and then compared and contrasted. Four studies related to the final research question (all qualitative).

### Data extraction and synthesis

Due to the differing nature of types of evidence collated in the review, it was necessary to extract different types of data. For quantitative studies rates of access, odds ratios, relative risks or proportions were extracted, whereas for qualitative studies main themes and interviewee quotes were extracted. Information relating to study characteristics was extracted for all studies (geographical location, number of participants, number of Pakistani female participants, age range, aims of the study, the target sample and research method). For qualitative studies that had conducted research with people from a range of ethnic groups, data relating to Pakistani women were extracted if possible.

## Results

### How does the usage of mental health services for Pakistani women in the UK compare with women of other ethnic groups?

Ten quantitative papers provided data that were relevant to this research question. Seven related to usage rates of inpatient services. One provided access rates to outpatient services, one reported on consultations with doctors for stress‐related or emotional problems, and one provided both rates of access for outpatient services, and consultations with general practitioners (GPs) (see Table 2).

**Table 2:** Summary of studies providing data on rates of usage of mental health services (*n* = 10)

Author (date)	Location	Sample size (age)	Pakistani women *N* (%)	Aims	Sample	Research method
Care Quality Commission & National Mental Health Development Unit (2011)	England and Wales	32,799 (all ages)	114 (0.3)	To obtain accurate figures relating to patients in mental health wards by ethnic group	Mental health unit inpatients and patients on Community Treatment Orders (CTOs)	Census
Care Quality Commission & National Mental Health Development Unit (2010)	England and Wales	31,786 (all ages)	110 (0.3)	To obtain accurate figures relating to patients in mental health wards by ethnic group	Mental health unit inpatients and patients on Community Treatment Orders (CTOs)	Census
Commission for Healthcare Audit and Inspection (2008)	England and Wales	31,020 (all ages)	121 (0.3)	To obtain accurate figures relating to patients in mental health wards by ethnic group	Mental health unit inpatients	Census
Commission for Healthcare Audit and Inspection (2007a)	England and Wales	31,187 (all ages)	85 (0.3)	To obtain accurate figures relating to patients in mental health wards by ethnic group	Mental health unit inpatients	Census
Commission for Healthcare Audit and Inspection (2007b)	England and Wales	32,023 (all ages)	104 (0.3)	To obtain accurate figures relating to patients in mental health wards by ethnic group	Mental health unit inpatients	Census
Commission for Healthcare Audit and Inspection (2005)	England and Wales	33,785 (all ages)	90 (0.3)	To obtain accurate figures relating to patients in mental health wards by ethnic group	Mental health unit inpatients	Census
Cochrane (1977)	England and Wales	N/R^a^ (15+)	N/R^a^	To report admissions to mental hospitals in 1971, by country of birth	Admissions to mental health hospitals in 1971	Survey
Glover and Evison (2009)	England	N/R^a^ (18–64)	N/R^a^	To examine the extent to which IAPT services have been used for ethnic minority groups	Patients using Crisis Resolution Home Treatment, Early Intervention, Assertive Outreach and IAPT services	Survey
Lloyd and Fuller (2002)	England	4281 (16–74)	387 (8.0)	To investigate the differences in mental health service use between ethnic groups	Household residents (sampled from The Health Surveys for England 1998 and 1999) with ethnic minority boost sample	Survey
Bajekal (2001)	England	16484 (16–74)	1,028 (6.2)	To investigate the differences in health service use and prescribed medicines between ethnic groups	Household residents, with ethnic minority boost sample	Survey

a Not reported.

For the papers that were included, there were differences in the way that rates could be interpreted. The Count me in Censuses (which accounted for six of seven papers reporting on access to inpatient services) provided counts of people who were using mental health inpatient services on census day (31st March). This differed from the paper by Glover and Evison (2009), which provided data on access to mental health outpatient services over 12 months. Both used NHS administrative data. The community surveys [(Health Survey for England 1999 and Ethnic Minority Psychiatric Illness Rates in the Community (EMPIRIC)] provided figures for consultations with GPs for mental illness based on participant self‐report and related to the previous 6 (EMPIRIC) or 12 (HSE 1999) months.

#### Inpatient services

The Count Me In Census of inpatients in mental health units in 2009 and 2010 (Care Quality Commission & National Mental Health Development Unit, 2010, 2011), estimated that the age‐ and sex‐standardised rates for admission for Pakistani women (2009, standardised ratio [SR] = 65, confidence interval [CI] = 53–79; 2010, SR = 70, CI = 57–84) were less than for white British women (2009, SR = 94, CI = 93–96; 2010, SR = 95, CI = 93–96) and less than the average rates (100) for women. However, the results from the years 2005 to 2008 suggested that the rates of admission for Pakistani women were no different from the average. Cochrane's study (1977) showed lower rates of admission for Pakistani (defined as born in Pakistani) women (374 per 100,000 – the lowest admission rate out of any country of birth).

#### Outpatient services

Only two papers provided rates of usage of outpatient services. One was a nationally representative English community survey (Ethnic Minority Psychiatric Rates in the Community (EMPIRIC), Lloyd & Fuller 2002), which showed no differences in the percentages of Pakistani women and white women who had seen a Community Psychiatric Nurse (1%) in the preceding 6 months, nor in the percentages that had seen a counsellor or psychologist within the same timeframe (2% for Pakistani women vs. 3% for white women).

The other paper (Glover & Evison 2009) provided rates of access to the following NHS services: Crisis Resolution Home Treatment (CRHT), Early Intervention (EI), Assertive Outreach (AO), and Improving Access to Psychological Therapies (IAPT) Services. The rates were provided for those aged 18–64 years, and used 2007 Office of National Statistics (ONS) Mid‐Year Estimates to standardise for age, although data were collected between March 2008 and March 2009. Compared with white British women's use of CRHT services (89.2/100,000, CI = 87.6–90.9), Pakistani women had lower rates of use (66.5/100,000, CI = 57.2–77.0). AO rates were similar for Pakistani (29.8/100 K, CI = 23.6–37.0) and white British women (36.5/100 K, CI = 35.5–37.6). However, Pakistani women had higher usage of EI services (109.5/100 K CI = 95.2–125.3) compared to white British women (76.4/100 K CI = 74.2–78.8). Pakistani women were less likely to be referred to IAPT services (212.7/100K, CI 161.5–275) compared with white British women (457/100K, CI = 444.8–469.5), and to receive treatment from IAPT services (165/100K, CI = 120.4–220.8 vs. 296.7/100K, CI = 286.9–306.8 for white British women). The authors also calculated the expected rates of referral and entry given the rates of psychiatric illness from the EMPIRIC survey (Sproston & Nazroo 2002) and concluded that referral and entry rates to treatment for Pakistani women were less than would be expected from the prevalence rates for common mental disorder.

#### Consultation of GP for mental illness

According to the estimates from EMPIRIC (Lloyd & Fuller 2002), Pakistani women were less likely to have consulted a doctor for emotional or stress‐related problems (relative risk [RR] = 0.60, SE = 0.16) compared to white women (RR = 1). However, the Health Survey for England 1999 (Bajekal 2001) concluded that when compared with women on average, there was no difference between the rates of Pakistani (standardised ratio [SR] = 1.21, SE = 0.11) and white women (SR = 1) who consulted the GP for being anxious or depressed. The opposite direction of effects in these two studies could be explained by the question wording. The EMPIRIC question asked whether the *last* visit to the doctor was for a ‘stress‐related or emotional problem’ (Sproston & Nazroo 2002, p. 182), whereas the HSE 1999 asked about service use over the last 12 months. Therefore, Pakistani women were less likely to have most recently visited the GP about a mental illness, but over the last 12 months, there was no difference in their consultation rates compared to white women.

All of the papers that addressed this research question, with one exception (Glover & Evison 2009), did not adjust mental health service usage rates for the level of mental illness (nor for socioeconomic factors). These omissions mean that the ethnic differences in mental health service use may have been underestimated, especially since the rates of mental illness are higher for Pakistani women (Weich & McManus 2002) and they are more likely to live in poverty (Nandi & Platt 2010).

### What is the nature of Pakistani women's social networks and how does this compare with women from other ethnic groups?

The quantitative papers were comparative in nature, investigating social support and social involvement in relation to women of other ethnic groups. Three of the qualitative papers focussed on Pakistani participants only, and one sought the views of older people from ethnic minority groups (Table 3). Papers focussed on contact with family and friends (which gives an indication of network content), and social support (network function).

**Table 3:** Summary of studies relating to social networks of Pakistani women (*n* = 7)

Author (date)	Location	Sample size (age)	Pakistani women *N* (%)	Aims	Sample	Research method
Platt (2009)	England and Wales	10,028 (16–65)	414 (4.1)	To explore the extent to which social activity in England and Wales varies by ethnic group and whether risks of social isolation are higher for some groups than others	Household residents, with ethnic minority boost sample	Survey
Natarajan (2006)	England	10,114 (16+)	795 (7.9)	To explore the differences in general health, acute sickness, long‐standing illness, psychosocial measures (GHQ12 and perceived social support) and prescribed medicines by ethnicity	Household residents, with ethnic minority boost sample	Survey
Stansfeld and Sproston (2002)	England	4,281 (16–74)	387 (8.0)	To examine the levels of support across different ethnic groups and to investigate whether this contributes to differences in psychiatric morbidity	Household residents (sampled from The Health Surveys for England 1998 and 1999) with ethnic minority boost sample	Survey
Calderwood and Tait (2001)	England	16,484 (16–74)	1028 (6.2)	To explore the differences in self‐reported long‐standing illness and acute sickness, self‐assessed general health and two measures of psychosocial health, the GHQ12 and perceived social support	Household residents, with ethnic minority boost sample	Survey
Gask *et al*. (2011)	East Lancashire	15 (23–73)	15 (100)	To examine the processes involved in why and how British Pakistani women fail to recover from depression and remain persistently low in mood	Pakistani women living in the local area with a diagnosis of depression from their GP	Qualitative interview
Rodriguez (2007)	North London	10 (40–59)	10 (100)	To address the issue of migration as a factor of change in the gendered division between private and public spaces	Pakistani women living in the local area, originating from Punjab or Sindh metropoles of Pakistan, with secondary school education or higher	Qualitative interview
Campbell and McLean (2003)	South England	26 (15–66)	13 (50.0)	To examine potential obstacles for Pakistani people in England to participate in local initiatives to reduce health inequalities	Pakistani Kashmiri residents in the local area	Qualitative interview

#### Network content

Pakistani women's social networks consisted mainly of relatives and other Pakistani people. Stansfeld and Sproston (2002) found that Pakistani women were the most likely (out of all ethnic groups: white, Irish, black Caribbean, Indian, Bangladeshi and Pakistani) to have seen relatives in the past month [ratio of means (RoM) = 1.33, SE = 0.12], but they were the least likely to have seen friends in the past month (RoM = 0.46, SE = 0.07). Campbell and McLean's (2003) study found that participants preferred to make friends with other Pakistani or South Asian people. However, the extent to which this was a choice for women appeared to be constrained by two factors: first several of the Pakistani‐born women in the sample ‘lived in households in which women did not leave the home unaccompanied’ and, second, women who had poor English language skills ‘were limited in their interaction with non‐Pakistani people’ (Campbell & McLean 2003, p. 14).

A strong sense of social isolation emerged as a core theme in some papers. Gask *et al*. (2011) found that social isolation was a feature of the experiences of Pakistani depressed women. This was perhaps to be expected given the nature of the sample, but it was also a feature of Pakistani women's networks in non‐clinical samples. Platt (2009) using the data from the 2001 Citizenship Survey, found that 17% of Pakistani women (the highest of any ethnic group: white British, black Caribbean, black African, Indian, Bangladeshi and Pakistani) were classified as socially isolated (defined as receiving or making infrequent visits, going out infrequently and low contact with clubs and organisations).

Participants in Campbell and McLean's (2003) study spoke of how involvement in community organisations was seen as a ‘white thing’ and if they participated in such groups, they might be accused of ‘acting white’ (p. 17) by people from their own ethnic group. Pakistani‐born women were often ‘isolated from mainstream English life’ (2003, p. 14) and while they were aware of the existence of women's groups and English classes, they rarely attended them. This was in contrast to younger England‐born Pakistani women who were more likely to be involved in community groups. Rodriguez (2007) reported that Pakistani women attended community centres and had built ‘social women‐centred (sic) networks’ (2007, p. 106). However, this study consisted of Pakistani women born in the Punjab or Sindh metropolitan areas, with high levels of education sampled in ‘mixed British and immigrant neighbourhoods’ (2007, p. 98), compared to the Pakistani Kashmiri women sampled by Campbell and McLean (2003) who were living in deprived (in the lowest quintile), multi‐ethnic neighbourhoods (at least 30% Pakistani), which may account for the difference in these findings.

#### Network function

The Health Survey for England (HSE) 1999 (Calderwood & Tait, 2001) found that 27% of Pakistani women perceived a severe lack of social support compared to 11% in the general population [odds ratio (OR) = 2.28, SE = 0.23]. Similar results were reported in Natarajan's paper (2006) using the HSE 2004 (Sproston & Mindell, 2006) where 30% of Pakistani women perceived a severe lack of social support compared to 11% in the general population (OR = 2.47, SE = 0.33).

Stansfeld and Sproston's paper (2002) found that Pakistani women reported high negative aspects of support (RR = 1.35, SE = 0.11) compared to white women (RR = 1). However, there were no differences in the perceived levels of low emotional and confiding support between Pakistani women (RR = 0.95, SE = 0.13) and white women (RR = 1). This could have been a result of the way emotional support was measured; it only related to the support received from a nominated closest person, whereas in the HSEs 1999 and 2004 social support related to all family and friends.

One study highlighted the importance of the (extended) family as a source of support, advice and care, and in some cases family were the only source of support available to Pakistani women, especially those who were born in Pakistan (Campbell & McLean 2003).

### What are the reasons for the mental health service utilisation patterns of Pakistani women? Are social networks implicated?

All the papers for this research question were qualitative in nature (Table 4). It was not the aim of any of the studies to investigate the association between social networks and usage of mental health services. However, there were indications that social networks in the form of family and close relatives could influence decisions to seek mental healthcare.

**Table 4:** Summary of studies relating to reasons for patterns of usage of mental health services (*n* = 4)

Author (date)	Location	Sample size (age)	Pakistani women *N* (%)	Aims	Sample	Research method
Wood (2011)	Leeds	5 (20–29)	4 (80.0)	To investigate how South Asian women understand and make sense of their experiences of self harm, and how they perceive support services	South Asian women aged between 18 and 40 with experience of self‐harm, educated in Britain in the local area	Qualitative interview
Chew‐Graham *et al*. (2002)	Salford and Trafford, Manchester	29 (17–50)	18 (62.1)	To investigate the self‐reported needs of South Asian women suffering mental illness which may lead to suicide and self‐harm	Attenders of existing South Asian Women's groups in the local area	Focus groups
Grewal and Lloyd (2002)	England	116 (25–50)	11 (9.5)	To examine respondents’ accounts of their pathways to mental health services	Purposive sample from EMPIRIC respondents	Qualitative interview
Cinnirella and Loewenthal (1999)	South East England, London and Midlands	52 (N/R^a^)	13 (25.0)	To investigate the degree to which beliefs about religion intertwine with lay beliefs about depression and schizophrenia	Pakistani Muslim, white Catholic, black African and Afro‐Caribbean Christian and white Orthodox Jewish women living in the specified local areas	Qualitative interview

a Not reported.

#### Coping alone as a result of the stigma of mental illness

All papers found that women felt they had to cope alone with mental illness. In three of four papers, one of the reasons for this was the stigma associated with having and speaking about mental illness, and this was argued to be directly linked to Pakistani culture: family and community members were seen as sources of stigmatising attitudes. The findings indicated that keeping problems to one's self was often a coerced choice, and one paper (Cinnirella & Loewenthal 1999) found that there were strong beliefs among participants that problems should be kept private within the family. The fear of being gossiped about was a strong theme in the focus groups conducted by Chew‐Graham *et al*. (2002) and the way in which this could ruin one's reputation was commented on by Cinnirella and Loewenthal (1999). None of the papers were comparative in nature, therefore the levels of stigma for Pakistani women could not be compared with women from other ethnic groups.

#### Preference for, but problems with, Pakistani health professionals

There was a clear contradiction evident in three of the four papers: Pakistani women preferred to see someone of their own ethnic group so that their problems could be understood appropriately; all of the women that took part in interviews in Cinnirella and Loewenthal's paper (1999) stated that they would prefer to see a Pakistani Muslim professional. However, women were also mistrustful of consulting health professionals from their own community due to fear of disclosure to family members and other people in the community. Only one paper found that the reason for wanting to see a professional of the same background was due to ‘mainstream service providers [who] were usually white’ (Chew‐Graham *et al*. 2002, p. 344) potentially having fixed views about the Pakistani community and displaying racism.

#### Language barriers

Two papers found that lack of English language skills affected access to, or experience of, services (Chew‐Graham *et al*. 2002, Grewal & Lloyd 2002). In particular, there was a sense that lack of English could impact negatively on knowledge of available services (Chew‐Graham *et al*. 2002) and on the quality of services received, if they were provided via an interpreter, as patients could not communicate directly with health professionals (Grewal & Lloyd 2002). Only one paper made reference to the lack of knowledge about mental health services among Pakistani women (Grewal & Lloyd 2002).

## Discussion

The review set out to answer whether the usage of mental health services differed between Pakistani women and women of other ethnic groups in the UK, the nature of Pakistani women's networks compared with women of other ethnic groups, and whether social networks were involved in seeking help for mental illness.

### Pakistani women are less likely to use specialist mental health services

The review provided evidence that rates of access to inpatient service are lower for Pakistani women than for white British women. Unfortunately, many of the studies did not take into account the level of mental illness nor did they adjust rates for important socioeconomic factors which are likely to impact on the access that people have to services (Goddard & Smith, 2001). Rates were adjusted for mental illness in relation to outpatient services in Glover and Evison's report (2009) and the authors suggest that rates of access to services were lower than would be expected for IAPT services. Rates of access to Early Intervention services for Pakistani women were higher than for white British women perhaps reflecting slightly higher rates of psychosis in older Pakistani women aged 55–74 years (Weich *et al*. 2004). Pakistani women's GP consultation rates for mental illness were about the same as for white women, which is surprising given that Pakistani women have higher GP consultation rates than most other ethnic groups (Nazroo *et al*. 2009).

Current UK mental health policy does not aim to directly tackle these ethnic inequalities in use of mental health services, although ‘under‐representation of Asian women receiving support from mental health services’ (Her Majesty's Government and Department of Health 2011, p. 26) has been highlighted as a reason for disaggregating outcome indicators provided by NHS England. Unfortunately, this has not happened; the latest figures for 2013/2014 are disaggregated by ethnicity (with Pakistani as a separate ethnic group), but not by gender (Community and Mental Health Team: Health and Social Care Information Centre, 2013b). NHS England have attempted to provide access rates to IAPT by ethnic group (Community and Mental Health team: Health and Social Care Information Centre, 2014b) and gender, but again this fell short of what was required, as figures were not age standardised nor adjusted for levels of mental illness. Further, ethnic group was not recorded for 28.5% of women.

### Pakistani women have high levels of social isolation

In comparison to other women, Pakistani women's networks were more likely to consist of a high number of relatives rather than friends. Pakistani women also had limited social interaction with people who were not Pakistani and those who were not part of their family or community. They exhibited low involvement in community organisations and clubs. Pakistani women's networks showed high levels of lack of social support and high negative aspects of networks compared to white British women.

Only one study (Campbell & McLean 2003) differentiated between women born in Pakistan and those born in England and how this might affect the nature of their social networks. They found that Pakistani‐born women's networks contained less interaction with people whose ethnicity was not Pakistani.

### Stigma towards mental illness and services within social networks

The studies reviewed showed that Pakistani women felt they had to cope alone with mental illness. There was an indication that social networks influenced attitudes towards mental health services and the course of action that Pakistani women chose to take. These negative attitudes about having mental illness were also evident in wider community networks. However, there were not any studies that provided information about the exact elements (e.g. spouse, wider family, friends) of social networks that displayed negative views about mental illness and mental health services. Previous studies in the United States suggest that increased contact with relatives can decrease mental health service use (Sherbourne 1988, Maulik *et al*. 2009), and increased support from friends has the potential to increase usage of mental health services (Vera *et al*. 1998). Hence, the type of contacts that are available in support networks can differentially influence usage of mental health services. This could be related to family members being more likely to feel the effects of stigmatising attitudes towards having a family member with mental illness; these effects may be felt less by friends.

One deterrent to accessing services was the fear that professionals of the same ethnic group would leak information to people that women knew. This was related to the stigma associated with having and talking about a mental illness. This is not something which is specific to Pakistani women. Rather it is a common feature among people suffering from mental distress (Thornicroft 2006), but there is the possibility that the level of stigma felt by Pakistani women may act as a greater deterrent to accessing services than for women from other ethnic groups. However, the levels of stigma by ethnicity could not be investigated in the review, because none of the papers commenting on stigma were comparative in nature. There are not any large survey data sets in the UK that have collected levels of felt stigma between ethnic groups, and it did not form a substantial part of the research programme undertaken by Time to Change England (cf. (Corker *et al*. 2013). However, there is evidence from other countries that for some ethnic minority groups, the stigma surrounding mental health‐related problems might be a greater deterrent to seeking help than for white majority populations (Pescosolido *et al*. 2013, Clement *et al*. 2015).

### Inadequacy of NHS services for Pakistani women

It was evident that some Pakistani women have language difficulties which impede their abilities to gain access to services, and can also have a negative effect on the experience of services. Only one study linked women's lack of willingness to access services due to racism from white health professionals. This may have been because none of the research studies were designed to specifically ask about racism with reference to health services. There were no studies looking at access to mental health services in the voluntary sector; given the results of the review that Pakistani women are overall less likely to access NHS mental health services, it is possible that the voluntary sector is a more likely route for gaining access to services.

### Strengths and limitations of the review

This is the first review to the authors’ knowledge that has been conducted on this ethnic group in the UK. Due to the very specific nature of the research questions, we were able to provide coherent answers in relation to Pakistani women in the UK. The findings of the results relating to mental health service usage and the quantitative studies about social support are generalisable to the population of England (or UK). The evidence obtained from the synthesis of the qualitative studies may not be generalisable to the wider population, but it is encouraging that many similar themes were extracted from these studies. In particular, there were very few Pakistani women (between 4 and 18) in the research studies that answered the third research question (whether social networks were involved the help‐seeking and access process for Pakistani women). Hence, the results for this research question are limited and should be viewed with caution. This also highlights the lack of studies in the UK that have sought to determine the influence of social networks on mental health service use, for both Pakistani women, and for women more generally. In addition, the use of the category ‘Pakistani’ is not without problems; the term must not be assumed to represent a homogenous group of women with identical backgrounds and experiences. However, the current ethnic categories that are used in research studies and national statistics are the only ones available for highlighting ethnic inequalities.

Many of the identified papers were excluded from the review due to their inapplicability to the research questions and a relatively small number (*n* = 9) were excluded due to methodological limitations at the critical appraisal stage. This is perhaps in contrast to other systematic reviews that excluded large numbers of papers due to poor quality (Morgan *et al*. 2013). This reflects the narrow nature of the topic and the lack of appropriate use of ethnic categories in previous research. Indeed a large number (*n* = 54) of studies were excluded because they did not analyse Pakistani women as a unique category, but chose to subsume Pakistani women into the broader category of South Asian women.

## Conclusions

Pakistani women are at a considerable disadvantage in gaining access to and using statutory mental health services, compared with white women. The review shows the importance of analysing Pakistani women separately from Indian and Bangladeshi women: future research and Department of Health published figures, should report and analyse Pakistani women as a separate group in order to provide accurate information on usage of mental health services. Current figures provided by the Department of Health via NHS England are not sufficient to monitor the differences in usage of mental health services between women of different ethnic groups, thereby preventing researchers to determine the equality or otherwise of usage of mental health services, on the grounds of ethnicity. Further, the review showed that Pakistani women tend to be socially isolated and have networks which display high levels of stigma towards mental illness and usage of mental health services. However, the review did not find any studies looking at the levels of stigma of mental illness between ethnic groups. Understanding Society, the UK's largest household longitudinal survey with an ethnic minority boost sample, is one survey that could be targeted by interested researchers, for inclusion of a survey module on mental health stigma, in order to establish if there are ethnic differences in mental illness stigma.

## Conflict of interests statement

All authors declare that they have no conflicts of interests.
